# Indoleamine 2, 3 Dioxygenase 1 Impairs Chondrogenic Differentiation of Mesenchymal Stem Cells in the Joint of Osteoarthritis Mice Model

**DOI:** 10.3389/fimmu.2021.781185

**Published:** 2021-12-08

**Authors:** Murad Alahdal, Rongxiang Huang, Li Duan, Deng Zhiqin, Ouyang Hongwei, Wencui Li, Daping Wang

**Affiliations:** ^1^ Hand and Foot Surgery Department, Shenzhen Second People’s Hospital (The First Hospital Affiliated to Shenzhen University), Shenzhen, China; ^2^ Shenzhen Key Laboratory of Tissue Engineering, Shenzhen Laboratory of Digital Orthopedic Engineering, Guangdong Provincial Research Center for Artificial Intelligence and Digital Orthopedic Technology, Shenzhen Second People’s Hospital (The First Hospital Affiliated to Shenzhen University, Health Science Center), Shenzhen, China; ^3^ Dr. Li Dak Sum & Yip Yio Chin Center for Stem Cells and Regenerative Medicine, Zhejiang University School of Medicine, Hangzhou, China

**Keywords:** osteoarthritis, cartilage regeneration, IDO1 inhibitor, MSCs, miR-122-5p

## Abstract

Osteoarthritis (OA) is a serious joint inflammation that leads to cartilage degeneration and joint dysfunction. Mesenchymal stem cells (MSCs) are used as a cell-based therapy that showed promising results in promoting cartilage repair. However, recent studies and clinical trials explored unsatisfied outcomes because of slow chondrogenic differentiation and increased calcification without clear reasons. Here, we report that the overexpression of indoleamine 2,3 dioxygenase 1 (IDO1) in the synovial fluid of OA patients impairs chondrogenic differentiation of MSCs in the joint of the OA mice model. The effect of MSCs mixed with IDO1 inhibitor on the cartilage regeneration was tested compared to MSCs mixed with IDO1 in the OA animal model. Further, the mechanism exploring the effect of IDO1 on chondrogenic differentiation was investigated. Subsequently, miRNA transcriptome sequencing was performed for MSCs cocultured with IDO1, and then TargetScan was used to verify the target of miR-122-5p in the SF-MSCs. Interestingly, we found that MSCs mixed with IDO1 inhibitor showed a significant performance to promote cartilage regeneration in the OA animal model, while MSCs mixed with IDO1 failed to stimulate cartilage regeneration. Importantly, the overexpression of IDO1 showed significant inhibition to Sox9 and Collagen type II (COL2A1) through activating the expression of β-catenin, since inhibiting of IDO1 significantly promoted chondrogenic signaling of MSCs (Sox9, COL2A1, Aggrecan). Further, miRNA transcriptome sequencing of SF-MSCs that treated with IDO1 showed significant downregulation of miR-122-5p which perfectly targets Wnt1. The expression of Wnt1 was noticed high when IDO1 was overexpressed. In summary, our results suggest that IDO1 overexpression in the synovial fluid of OA patients impairs chondrogenic differentiation of MSCs and cartilage regeneration through downregulation of miR-122-5p that activates the Wnt1/β-catenin pathway.

**Graphical Abstract:**
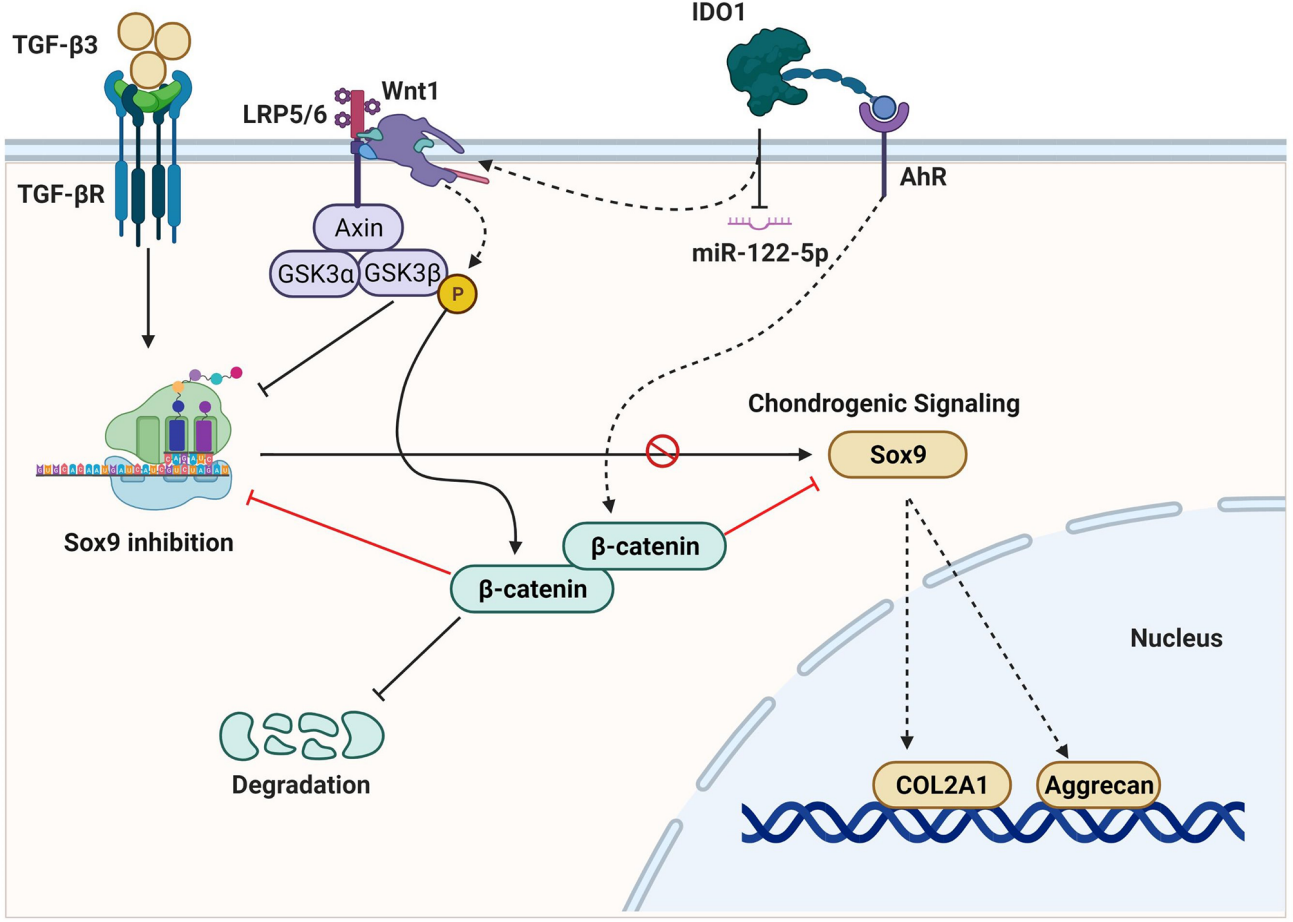


## Introduction

Osteoarthritis (OA) is an articular cartilage degenerative disease that causes serious dysfunction of the joint (1). The global incidence of OA increases rapidly and may become the largest cause of disability worldwide by 2030 (2, 3). Up to date, the novel strategy to reduce the progression of OA depends on eliminating articular cartilage loss and enhancing cartilage regeneration (4). Thus, clinical attempts work to slow down cartilage degeneration and enhance cartilage repair with various strategies such as modification of cytokines, chemokines, and enzymes (5). However, the efficiency of pushing forward cartilage regeneration is still unsatisfactory and poorly understood (6). The use of mesenchymal stem cells (MSCs) transplantation for cartilage repair and OA treatment was reported by several studies and clinical trials (5, 7, 8). But recent studies reported unsatisfactory outcomes including calcification (9) and other challenges that slow down the regeneration of cartilage and completely inhibit cartilage repair without clear reasons. Nevertheless, the effect of biological mediators in the joint cavity on the chondrogenic function of MSCs remains to be studied. We noticed that high levels of indoleamine 2,3 dioxygenase 1 (IDO1) were reported in the synovial fluid of OA patients (10–12).

The activity of IDO1 contributes to tryptophan catabolism and the induction of arthritis. However, the crucial role IDO1 plays in the OA disease remains controversial. Some reports concluded that the blocking of IDO1 increased the inflammatory responses in the arthritis animal model (13, 14). But other clinical studies suggested that the overexpression of IDO1 could play a role in the stiffness of joints and may increase the severity of arthritis (12, 15). Further, there is a previous study that reported that the inhibition of IDO reduced the inflammations in arthritis animal models (16, 17). However, the effect of IDO1 overexpression on the cartilage regeneration and chondrogenic differentiation of MSCs hasn’t been reported. We supposed that IDO1 overexpression in the synovial fluid of OA patients could play a role in the prevention of MSCs’ differentiation to mature chondrocytes because of the tryptophan degradation effect. Furthermore, our results explored a potential role of IDO1 in the low efficiency of chondrogenic differentiation of MSCs through its downregulation to miR-122-5p, which activates Wnt1/β-catenin pathway. The activation of the Wnt1/β-catenin signaling reduces the expression of Sox9 and COL2A1. Moreover, MSCs mixed with IDO1 inhibitor successfully promoted cartilage regeneration in the animal model. Therefore, this study reports for the first time a new function of IDO1 in the joint on OA animal model.

## Materials and Methods

### The Establishment of the OA Animal Model

Forty female Wistar rats were purchased from Beijing Vital River Laboratory Animal Technological Company (Beijing, China) at 14–15 weeks of age (300–350 g). Animals were housed in plastic cages under pathogen-free conditions. The experiments were performed in accordance with the National Institute of Health Guide for the Care and Use of Laboratory Animals and approved by the ethical committee of Shenzhen University. Briefly, Wistar rats were anesthetized with isoflurane by healing, and then subjected to a modified surgical procedure as follows: (1) The connective tissue between the knee patellar ligament and the medial collateral ligament was cut. Then the joint capsule was exposed. (2) The joint capsule was opened, then the dislocation of the patella to the outside, and the exposing of the anterior cruciate ligament and cutting out were performed. (3) The ligament of the medial meniscus platform was then cut out, and the anterior angle of the medial meniscus was removed posteriorly. (4) Layer-by-layer suture and disinfection followed. Sham group: only the skin and joint cavity were opened; the incision was rinsed with normal saline and then sutured. Next, rats that underwent meniscectomy were divided into three groups; each group had 10 rats. The normal control group also included 10 rats. The total groups were four groups. (5) OA model establishment confirmation: serum samples from all groups were collected to test inflammatory cytokines IL-1β, TNF-α, and IFN-γ. Later, the analysis of inflammatory cytokines was performed using ELISA kits. Briefly, from the tail vein 1 ml of blood was obtained and transferred to 5 ml tubes. After complete agglutination, blood samples were centrifuged at 2,000 rpm/10 min to isolate serum. Mouse IL-1β, TNF-α, and IFN-γ ELISA kits were purchased from Multi-sciences, China. The experiments were performed according to the manufacturer instructions. Results were visualized by GraphPad Prism 9.

### The Effect of IDO1 on the Cartilage Regeneration Based on MSCs Therapy in OA Model

Fresh SF-MSCs derived from rats were purchased from Cyagen, USA. SF-MSCs were mixed with 50 ng/ml IDO1 inhibitor (Epacadostat) (Selleck, USA) compared to 50 ng/ml IDO1 (Cusabio, USA). Then, rats were divided into four groups; the rats of the first group were injected with 100 µl SF-MSCs (1×10^6^ cell/µl) + Epacadostat; the rats of the second group were injected with 100 µl SF-MSCs (1×10^6^ cell/µl) +IDO1; OA positive control group was injected with 100 µl PBS; the fourth group was the normal control group. The injection was repeated every 2 weeks for 2 months.

### Histological Analysis

In order to confirm the establishment of the OA model 1 month post the operation and to test the effect of injected regimes on the cartilage tissue, first, three rats from every group were selected randomly and sacrificed. The knee joints were fixed using 4% paraformaldehyde (Thermofisher, China). Next, joints were embedded in the paraffin and sectioned into 5 μm thick, and then deparaffinized, hydrated, and stained with Hematoxylin and Eosin (HE) as described in the previous study (18). Two months later, all rats were sacrificed, and then knee joints and synovial fluid samples were collected in special tubes that were supplemented with anti-agglutination. The HE staining and toluidine blue staining were performed. The histological sections were sent to a pathologist for confirmation of the diagnosis and interpretation of the results.

### The Isolation and Characterization of Human SF-MSCs Through Epitope Profiling

A synovial fluid (SF) sample was obtained from the residue of the non-OA patient (female, 64 years old) that underwent a joint replacement surgery. SF sample was transferred to the laboratory and then centrifuged at 2,000 rpm/5 min. The supernatant was kept in a separate tube, and the residue was washed twice using DMEM medium. Then, it was cultured in the hMSCs special medium (MeseGro + supplements) with 10% fetal bovine serum (FBS) and 1% penicillin and streptomycin. Five days later, cells were refreshed with the new medium. The third generation of isolated cells was subjected to characterization experiments. Briefly, 5 million of isolated cells were divided into test groups and control groups. FC blocker was applied 15 min on ice. Then, FITC-anti-human anti-CD73, PE- anti-human anti-CD105, APC- anti-human anti-CD166, PerCP- anti-human anti-CD34, FITC- anti-human anti-CD90, and PE- anti-human anti-CD45 (Biolegend, China) were used for staining of surface markers for 30 min on ice. Later, cells were washed and visualized using Beckman Coulter (Navios, USA). Finally, data were analyzed and presented using FlowJo v10 software.

### The Effect of IDO1 on the Chondrogenic Stimulation of SF-MSCs

The effect of IDO1 on the chondrogenic stimulation of SF-MSCs compared to IDO1 inhibitor (Epacadostat) was assessed by coculturing SF-MSCs with 50 ng/ml of Epacadostat compared to 50 ng/ml of IDO1 for 6 days. Briefly, total RNA was extracted using TRIzol (Ambion, USA) according to the kit instructions. cDNA library was built using a cDNA construction kit (Takara, China) according to the kit instructions. The primers of COL2A1, Sox9, Ihh, and Aggrecan were predicted using NCBI gene ID in the primerbanck web (https://pga.mgh.harvard.edu/primerbank/); see **Supplementary Table 1**. Then, forward and reverse primers were designed by Shanghai Gene Pharma (Genepharma, China). The quantitative amplification of tested genes was detected using SYBR Green kits (Takara, China) and tested by qPCR (Biosystem 7500, USA). Results were visualized using ^Δ^CT values that were validated by GAPDH control in the GraphPad Prism 9. Further, the accumulation of proteoglycan was tested by Alcian blue staining for MSCs cocultured with IDO1 compared to Epacadostat. Briefly, Alcian blue was implemented for 15–20 min, and then cells were washed three times with PBS. Cells were visualized using light microscopy on 40× magnification. Furthermore, the expression of COL2A1 and Sox9 was visualized using confocal laser microscopy. Briefly, cells were suspended in the PBS and fixed on the glass slides using cytospin centrifuge 1,500/5 min (Thermofisher, China). Fixed cells were subjected to BSA (0.05 g/ml) for 60 min. Then, cells were washed five times with PBS (5 min/time). Next, cells were incubated overnight at 4°C with anti-human anti-collagen type II (Bioss antibodies, China), and anti-human anti-Sox9 (Biorbyte, China). Later, cells were washed with PBS three times (5 min/time). Secondary antibody anti-mouse IgG was applied for 2 h. Then cells were washed again three times. After that, cells were incubated for 10 min with DAPI at 4°C. Finally, cells were washed five times with PBS and visualized using a confocal laser microscope (ZEIZZ LSM800, Germany).

### The Effect of IDO1 on the Apoptosis of Chondrocytes

In order to assess the effect of IDO1 on the chondrocytes, 1×10^6^ cells/well were cultured in a 96-well plastic plate. Then 50 ng/ml/well of IDO1 was added at different time points (24, 48, 72, and 96 h) compared to PBS. As well, the same experiment was repeated using Epacadostat 50 ng/ml/well compared to PBS. Cck8 buffer was added according to the instructions of the kit (Yeasen, USA). After 4 h, OD was measured by microplate reader at 450 nm.

To confirm the apoptotic effects of IDO1 on the chondrocytes, annexin-v assay was performed. Briefly, 1×10^5^ cells were cultured with 50 ng/ml IDO1 for 72 h. Later annexin-V-FITC and PI were applied according to the kit instructions (BioVision, Canada). Then, the apoptosis was visualized using confocal laser microscopy.

### The Molecular Effects of IDO1 Knockdown on the Chondrogenicity of SF-MSCs

IDO1 gene was knocked down using the siRNA transfection technique. Briefly, chondrogenic SF-MSCs were transfected with three different IDO1-siRNA models that were predicted by siRNA at WHITEHEAD (http://sirna.wi.mit.edu/home.php) (see **Supplementary Table 2**). Then, it was produced by Gene Pharma Company (Shanghai, China). The transfection was performed according to the kit instructions of lipofectamine2000 (Thermofisher Sscientific, China). Twenty-four hours later, total RNA was collected and cDNA transcription was performed using TaKaRa Taq DNA Polymerase kit. The performance of siRNA was tested by qPCR using the IDO1 primers listed in the **Supplementary Table 1**. The performance of IDO1-siRNA was validated by western blot using anti-human anti-IDO1 (Sinobiological, China) compared to anti-GAPDH. Next, the expression of β-catenin, Sox9, and Cola2 was assessed using western blot. Briefly, total protein extraction, BCA, and western blot were performed as the following: total protein was extracted using RIPA buffer (99 ul/1×10^6^ cells), and 1 ul of PMSF (proteinase inhibitor) was added. Cells were put on the ice for 30 min every (mix every 5 min). Then, cells were centrifuged at 12,000 rpm/10 min, and then the supernatant was transferred into a clean 1 ml Eppendorf tube. The concentration of total protein was evaluated using a BCA kit (Sigma, USA) according to the kit instructions. The protein samples were run on the 10% gel SDS-PAGE. Then, protein bands were transferred on the PVDF membrane using a Genscript transfer machine (Genscript, USA) for 12 min. Next, the transferred proteins were blocked using sheep milk for 1 h. After that, milk was washed three times using TBS 1× buffer every 5 min. Later, primary antibodies anti-human anti-Sox9, anti-COL2A1, anti-β-catenin, and anti-GAPDH (Abcam, USA) were added to the membranes overnight. Then, membranes were washed three time (one/5 min) using TBS buffer. Secondary anti-rabbit antibody IgG was added for 2 h. Then, it was washed three times as described above. Finally, protein bands were visualized and analyzed using ODYSSEY FC (Gene company, China).

### Testing the Effect of β-Catenin Knockdown on the Expression of Sox9 Under the Effect of IDO1

β-catenin was knocked down in the chondrogenic SF-MSCs using siRNA and lipofectamine2000; three different sequences for siRNA were predicted and designed by (Gene Pharma, China). Sense and anti-sense sequences were listed in **Supplementary Table 2**. The performance of siRNA activity was tested by qPCR as described above, and the used primers were listed in **Supplementary Table 1**. Then, cells with β-catenin knocked down (SF-MSCs -/- β-catenin) were cultured with IDO1 as described above. After that, these cells were tested by qPCR for Sox9 expression. The expression of Sox9 was also tested by immunofluorescence assay and flowcytometry as described above.

### The Assessment of β-Catenin Upstream Molecules Under the Effects of Epacadostat

As known, the upstream molecules of β-catenin in the Wnt pathway are GSK3α, GSK3β, and APC. These molecules have been tested under the effect of Epacadostat compared to IDO1 using qPCR; primers were listed in **Supplementary Table 1**. The analysis of GSK3β phosphorylation under the effect of Epacadostat compared to IDO1 was tested by immunofluorescence assay and western blot using GSK3β phospho9 antibody (Abcam, USA). Briefly, different concentrations of Epacadostat and IDO1 were tested in the culture of chondrogenic SF-MSCs (100 μg/ml and 50 μg/ml) to observe the expression changes of phosphorylated protein under IDO1 overexpression and inhibition. Later, the effect of Epacadostat and IDO1 on the phosphorylation of GSK3β was confirmed using flowcytometry.

### The Interaction Between Sox9 and β-Catenin

The interaction between Sox9 protein and β-catenin protein was analyzed by using two methods as the following: (1) Using bioinformatics: the protein sequence of Sox9 and β-catenin were used for predicting protein-protein interaction (PPI) using the following datasets (www.String-db.org and www.thebiogrid.org). Then bitmap images were extracted. Later, a colocalization assay was performed. Briefly, after treating chondrogenic SF-MSCs with Epacadostat compared to IDO1 for 3 days, cells were stained with anti-human anti-Sox9 red (Bioyt, China) and anti-human anti-β-catenin green (Abcam, USA). The analysis of protein overlapping was performed on confocal laser microscopy. Color intensity was analyzed and normalized using the correlation coefficient by GraphPad 9.

### The Analysis of Wnt1 and AhR Receptors Under the Effect of Epacadostat Compared to IDO1

Chondrogenic SF-MSCs were cultured with Epacadostat compared to IDO1 for 3 days. The expression of Wnt1 and the AhR genes was tested using qPCR. Primers are listed in **Supplementary Table 1**. The interaction between AhR receptor and β-catenin was predicted by bioinformatics (www.thebiogrid.org). These results were confirmed by confocal laser microscopy using anti-human anti-Wnt1 and anti-human anti-AhR (Abcam, USA).

### miRNA Transcriptome Sequencing

SF-MSCs have been cultured with IDO1 compared to normal control cells for 6 days, and then total RNA was collected using TRIZOL as described above. The RNA samples were shipped to Guangzhou Ruibo Biotechnology Co., Ltd for performing miRNA-Seq. The accession number is PRJNA758741; the data are available in the NCBI (https://www.ncbi.nlm.nih.gov/sra/PRJNA758741).

### Evaluation of the Levels of miR-122-5p in MSCs

The levels of miR-122-5p have been tested in the obtained total RNA using TaqMan MicroRNA Reverse Transcription kit (Thermo Fisher, USA). Only the mature miR-122-5p sequence was provided as 5’-UGGAGUGUGACAAUGGUGUUUG-3’. Synthetic RNA oligonucleotide, cel-miR-39-3p (Qiagen), was used for normalization of qPCR results. The supposed interaction between Wnt1 and miR-122-5p was predicted using miRanda website. Then, the expression of miR-122-5p was overexpressed and inhibited in the SF-MSCs using miR-122-5p mimics and miR-122-5p inhibitor in the presence of miRNA-NC (Genepharma, China). Next, the expression of Wnt1 was tested using qPCR and western blot.

### The Statistical Analysis

Statistical analysis was performed using IBM SPSS statistics 22 for at least three independent experiments. Statistical significance comparing two groups with parametric data was assessed by paired student’s t-test. Statistical analysis comparing multiple groups with parametric data was performed by one or two-way ANOVA. P<0.05 value was considered statistically significant. All data were presented as mean ± standard deviation.

## Results

The results showed that IDO1 plays a negative role in the synovial fluid of the OA animal model. It inhibits cartilage regeneration and MSCs-based therapy through downregulation of miR-122-5p, which activates the signaling of wnt1/β-catenin that significantly inhibited the expression of Sox9 and COL2A1. Further, the inhibition of IDO1 significantly promoted cartilage repair and chondrogenic differentiation of MSCs.

### IDO1 Impairs MSCs-Based Therapy and Cartilage Regeneration

**Figure 1:**
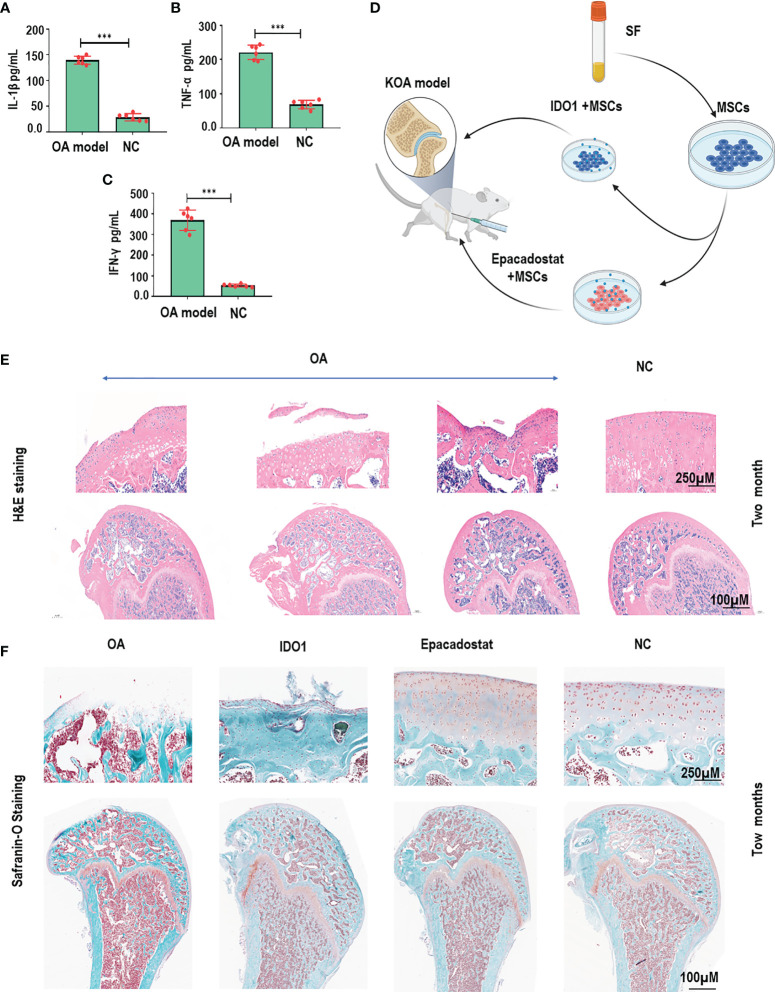
IDO1 inhibitor mixed with MSCs improved the efficacy of MSCs to promote cartilage regeneration in OA animal model. **(A)** The levels of IL-1β in the OA animal model after one month of model establishment compared to normal control. **(B)** The levels of TNF-α in the OA animal model after one month of model establishment compared to normal control. **(C)** The levels of IFN-γ in the OA animal model after one month of model establishment compared to normal control. **(D)** The schematic diagram presented the performed strategy for stimulated MSCs to be used in the cartilage repair for the OA model. **(E)** HE staining for knee joints of OA animal model after one month that presented the initiation of cartilage degeneration compared to normal control groups. **(F)** Safranin-O staining for the cartilage of OA model after two months of treatment with Epacadostat-treated MSCs, IDO1-treated cells, without treatment group, normal control group. ***p < 0.001 *vs* NC group.

In order to observe the effect of IDO1 blocking during MSCs transplantation on cartilage regeneration, the OA animal model was established as presented in **Supplementary Figure 1**. Results, as presented in **Figures 1A–C**, **E**, showed significant cartilage degeneration and increased inflammatory cytokines in the OA animal model compared to normal control. Two months of treatment with MSCs+Epacadostat compared to MSCs+IDO1 as presented in **Figure 1D** demonstrated that MSCs+Epacadostat significantly promoted cartilage regeneration as seen in **Figure 1F** and **Supplementary Figure 2A**. In contrast, MSCs+IDO1 didn’t show any improvement in the joint, but it presented an increased cartilage degeneration, suggesting that IDO1 impaired the differentiation of SF-MSCs and subsequently cartilage repair. Further, the magnification focused on the chondrocytes inside the cartilage tissue as presented in **Supplementary Figures 2B, C** showed matured chondrocytes and healthy cartilage in the group injected MSCs+Epacadostat. But in the group that was injected with MSCs+IDO1, the chondrocytes were showed apoptosis in the degenerative cartilage.

In order to confirm the apoptosis effects of IDO1 on the chondrocytes, Annexin-V/PI and cck8 assays were performed. Results elucidated that IDO1 significantly promoted the apoptosis of chondrocytes during 72 h of cultivation as seen in **Supplementary Figures 2C, D**. Therefore, these results confirmed that IDO1 impairs the chondrogenicity of MSCs and enhances cartilage degeneration.

### IDO1 Inhibits Chondrogenic Differentiation of SF-MSCs

**Figure 2:**
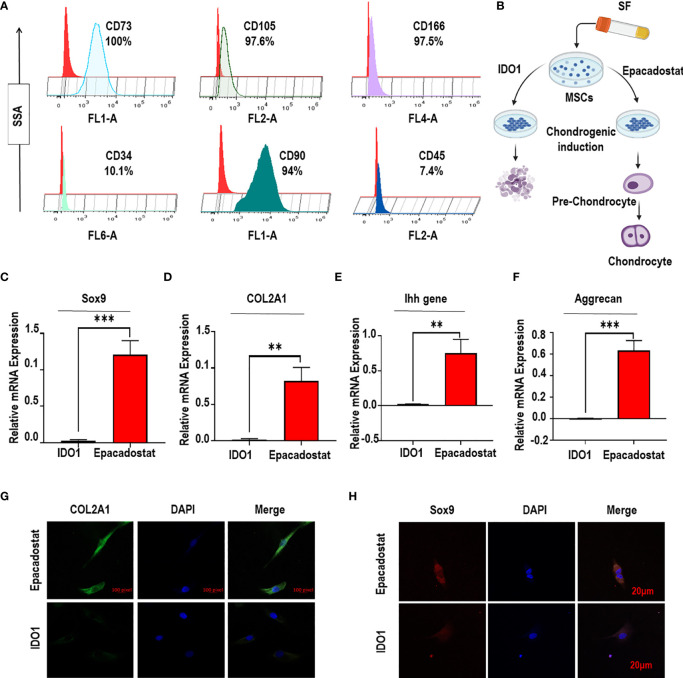
IDO1 decreases the expression of chondrogenic genes in the stimulated MSCs. **(A)** Flow cytometry surface epitope profiling of MSCs. **(B)** The effect of IDO1 compared to Epacadostat on the chondrogenic differentiation potency of MSCs. **(C)** The expression of Sox9 under the effect of IDO1 compared to Epacadostat. **(D)** The expression of COL2A1 under the effect of IDO1 compared to Epacadostat. **(E)** The expression of the Ihh gene under the effect of IDO1 compared to Epacadostat. **(F)** The expression of Aggrecan under the effect of IDO1 compared to Epacadostat. **(G)** The expression of COL2A1 under the effect Epacadostat using IF assay. **(H)** Epacadostat promotes Sox9 signaling compared to IDO1. ***p < 0.001 and **p < 0.01 *vs* NC group.

The characterization of MSCs as presented in **Figure 2A** showed typical surface epitopes of MSCs; strong positive of CD73, CD90, CD105, and CD166, while CD34, and CD45 were obviously very low. The coculture of SF-MSCs with IDO1 compared to Epacadostat showed that IDO1 significantly inhibited the expression of Sox9 (*p*<0.001), COL2A1 (*p*<0.01), Ihh gene (*p*<0.01), and Aggrecan (*p*<0.001) as presented in **Figures 2B–F** and **Supplementary Figure 3C**. Further, IDO1 inhibited the production of sulfated proteoglycan as seen in **Supplementary Figures 3A, B**, **D**. The differentiation of chondrogenic stimulated cells was increased by Epacadostat compared to IDO1. Further, the effect of IDO1 on the expression of Sox9 and COL2A1 in the stimulated MSCs was confirmed by confocal laser microscopy, as seen in **Figures 2G, H**. IDO1 significantly prevented the expression of COL2A1 and Sox9 in comparison to Epacadostat that enhances the expression of COL2A1 and Sox9 in the stimulated SF-MSCs. Furthermore, we noticed that IDO1 promoted the expression of β-catenin, as seen in **Supplementary Figure 3C**.

### IDO1 Knockdown Enhances the Expression of Sox9 in Stimulated MSCs Through Inhibiting the Expression of β-Catenin

**Figure 3:**
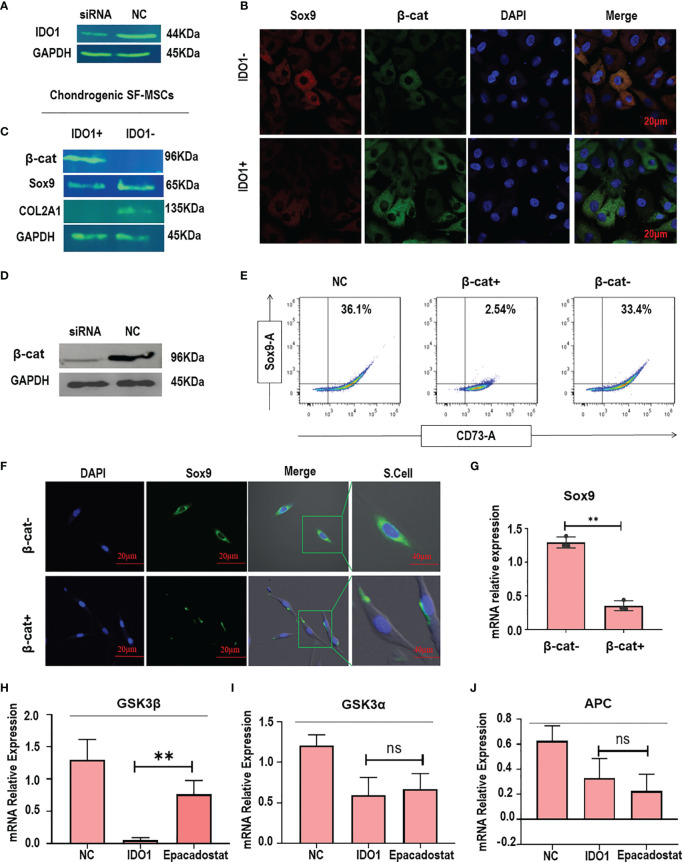
IDO1 regulates the expression of Sox9 through activating β-catenin in the MSCs. **(A)** Silencing of IDO1 using siRNA in the MSCs. **(B)** The immunofluorescence expression of Sox9 and β-catenin in the IDO1 silenced cells compared to IDO1-positive cells. **(C)** The expression of Sox9, COL2A1, and β-catenin by western blot in the IDO1 knocked cells compared to active IDO1. **(D)** The knocking down of β-catenin in the SF-MSCs using siRNA transfection. **(E)** The expression of Sox9 under β-catenin silencing in the SF-MSCs compared to active β-catenin using flow cytometry. **(F)** The expression of Sox9 under β-catenin silencing using confocal laser microscopy. **(G)** The expression of Sox9 under β-catenin silencing by qPCR. **(H)** The expression of GSK3β in the MSCs under the effect of IDO1 and Epacadostat compared to the NC group. **(I)** The expression of GSK3α in the MSCs under the effect of IDO1 and Epacadostat compared to the NC group. **(J)** The expression of APC in the MSCs under the effect of IDO1 and Epacadostat compared to the NC group. **p < 0.01 *vs* NC group. ns, no significance.

In order to explore the mechanism of the effect of IDO1 in the MSCs, IDO1 was knocked down as seen in **Figure 3A**. The knockdown of IDO1 showed a significant increase in the expression of Sox9 and decreased expression of β-catenin as presented in **Figures 3B, C**, indicating that IDO1 inhibits Sox9 and COL2A1 by increasing the expression of β-catenin. Further, the knocking down of β-catenin in the SF-MSCs as shown in **Figure 3D** promoted the expression of Sox9 in the stimulated MSCs even in the presence of IDO1, as presented in **Figures 3E–G**. These results obviously prove that IDO1 impairs chondrogenic signaling by increasing the expression of β-catenin.

### IDO1 Enhances the Expression of β-Catenin Through Downregulating GSK3β

In order to explore how IDO1 promotes the expression of β-catenin, the effect of IDO1 on the upstream molecules was tested. Results showed that IDO1 significantly reduced the expression of GSK3β (*p*<0.01), as presented in **Figure 3H**. Other tested upstream molecules didn’t show any significant change under the effect of IDO1 as seen in **Figures 3I, J**. Interestingly, the effect of IDO1 on the GSK3β expression has been reduced by Epacadostat. These results presented that the cross-talk between IDO1 and β-catenin in the MSCs is through GSK3β.

### The Phosphorylation of GSK3β in MSCs Mediated by IDO1

**Figure 4:**
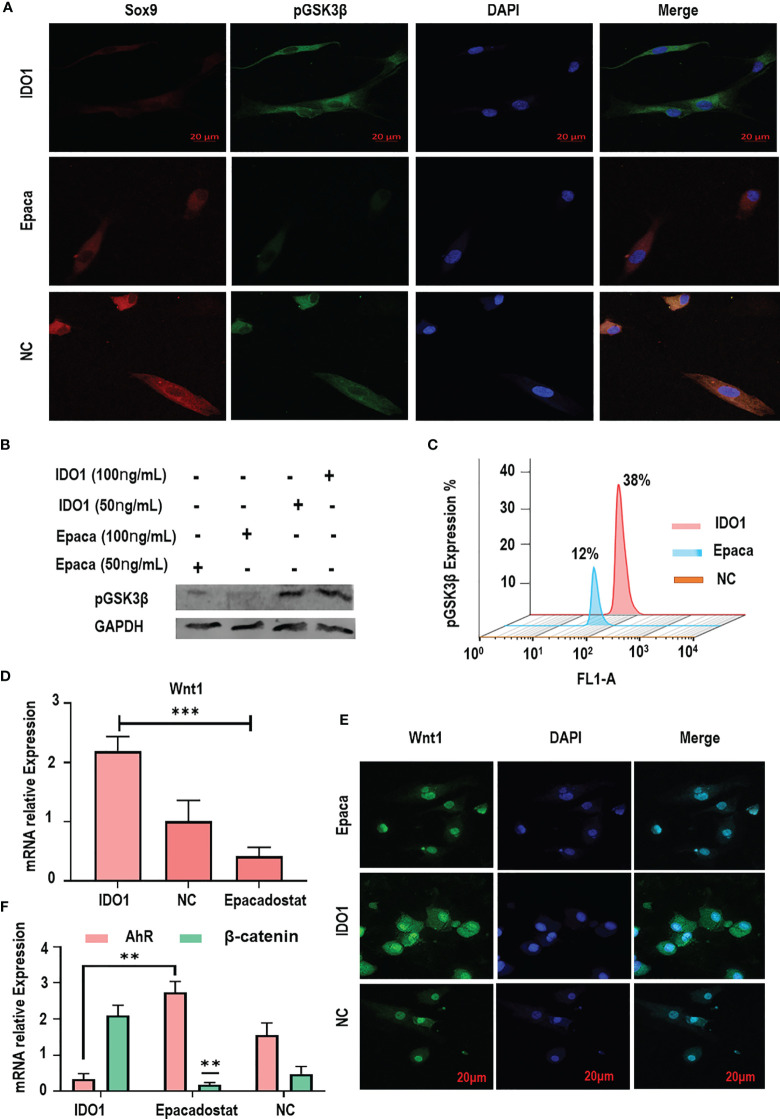
IDO1 decreases the expression of GSK3β in MSCs through inducing its phosphorylation. **(A)** The immunofluorescence expression of pGSK3β showed increased expression under IDO1 effects, while it was reduced by Epacadostat, suggesting the inhibition of GSK3β phosphorylation by Epacadostat. **(B)** The western blot expression of pGSK3β under the effect of IDO1 compared to Epacadostat in the MSCs; the effect of two different concentrations showed a change in the expression of phosphorylation protein. **(C)** The expression of pGSK3β under the effect of IDO1 compared to Epacadostat by flow cytometry. **(D)** The expression of Wnt1 in the MSCs under the effect of Epacadostat compared to IDO1. **(E)** The immunofluorescent expression of Wnt1 in the MSCs under the effect of Epacadostat compared to IDO1. **(F)** The expression of the AhR receptor and β-catenin in the MSCs under the effect of Epacadostat compared to IDO1. ***p < 0.001, and **p < 0.01.

To explore the mechanism by which IDO1 regulates the expression of GSK3β in the stimulated MSCs, the phosphorylation of GSK3β was tested. Results revealed that IDO1 significantly promoted GSK3β phosphorylation as shown in **Figures 4A–C**, while Epacadostat significantly inhibited the phosphorylation of GSK3β. Further, immunofluorescence assay showed that Epacadostat inhibited the expression of S9 phosphoprotein, while IDO1 enhanced the expression of phosphorylation protein as seen in **Figure 4A**. These results were confirmed by testing the different concentrations of Epacadostat compared to IDO1 as seen in **Figure 4B**. The increased concentration of IDO1 increased the expression of pGSK3β, while Epacadostat obviously inhibited the phosphorylation of GSK3β. The results of flow cytometry confirmed the same observations as presented in **Figure 4C**. Importantly, we revealed that the expression of Sox9 was enhanced when GSK3β phosphorylation was inhibited, and vice versa, as seen in **Figure 4A**.

### IDO1 Increased the Expression of Wnt1 While Downregulating the Expression of AhR

To elucidate how IDO1 promotes the phosphorylation of GSK3β, the expression of Wnt1 and AhR receptors was tested. Results presented that IDO1 significantly increased the expression of Wnt1 (*p*< 0.001), which promoted GSK3β phosphorylation as seen in **Figures 4D, E**. Meanwhile, the inhibition of IDO1 reduced the expression of Wnt1 significantly. In contrast, the expression of AhR receptor has been enhanced by Epacadostat significantly as presented in **Figure 4F** and **Supplementary Figure 4A**, while IDO1 strongly inhibited AhR. Bioinformatic analysis of AhR and β-catenin interaction as presented in **Supplementary Figure 4B** showed a direct interaction between AhR and β-catenin, which indicates that Epacadostat inhibits Wnt1 and enhances AhR expression, at the same time leading to downregulation of β-catenin and promoting Sox9. Thus, IDO1 upregulates the expression of Wnt1 to activate the phosphorylation of GSK3β in the MSCs.

### The Anti-Localization Effect of β-Catenin on Sox9 in MSCs

**Figure 5:**
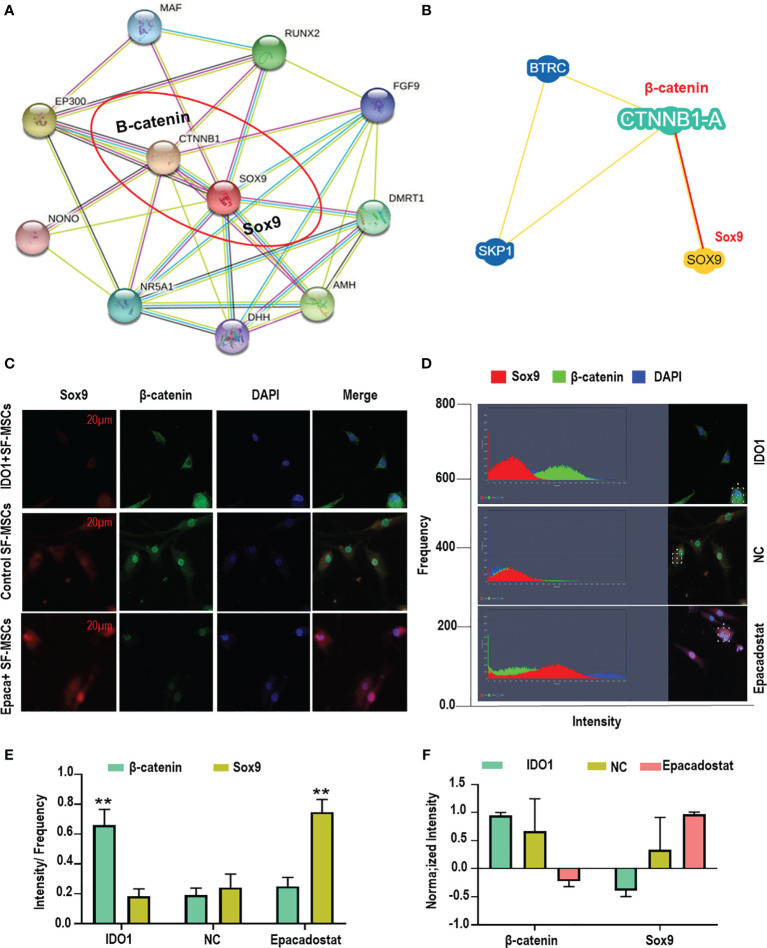
IDO1 induces the anti-localization interaction between β-catenin and Sox9 in the MSCs. **(A, B)** Bioinformatic analysis of P-P interaction between β-catenin and Sox9 using the database of String-db.org and thebiogrid.org. **(C)** Colocalization assay between β-catenin and Sox9 using immunofluorescence overlapping. **(D)** The analysis of expression intensity. **(E)** The calculation of intensity by frequency of every protein expression. **(F)** The normalizing intensity by calculating Pearson correlation. **p < 0.01 Epacadostat *vs* IDO1.

The molecular interaction between β-catenin and Sox9 was assessed using bioinformatics and colocalization assay to explore the mechanism of interaction between both proteins in the cell. The results presented that β-catenin directly interacts with Sox9 tail as presented in **Figures 5A, B** (https://version-11-5.string-db.org/cgi/network?networkId=bbfXNrQuYRTn / https://thebiogrid.org/98683/network/xenopus-laevis/ctnnb1-a.html). The analysis of colocalization assay elucidated that β-catenin reduces the expression of Sox9 as seen in **Figures 5C, D** and **Supplementary Figure 5A**, suggesting that β-catenin anti-localizes with Sox9 under the effect of IDO1. But Epacadostat inverted this effect significantly (*p* < 0.01), which promoted the expression of Sox9 through enhancing the degradation of β-catenin. The position of interaction between Sox9 and β-catenin seems to be around the nucleus but not inside the nucleus, which indicates that direct accumulation of β-catenin around the nucleus abolishes the function of Sox9. Hence, downregulation of β-catenin by Epacadostat enhanced the free expression of Sox9 in the SF-MSCs model. Statistical analysis of expression intensity confirmed the inverse relation between Sox9 expression and β-catenin as seen in **Figures 5E, F**. These results strongly proved that IDO1 activates the expression of Wnt1/β-catenin to impair the chondrogenic differentiation of MSCs. However, the molecular mechanism by which IDO1 mediated the activation of the Wnt1/β-catenin leading to inhibit Sox9 is unknown. Therefore, we have performed miRNA sequencing for MSCs under the effect of IDO1. The results are presented in the following section.

### IDO1 Downregulates miR-122-5p to Promote Wnt1 Activation and GSK3β Phosphorylation

**Figure 6:**
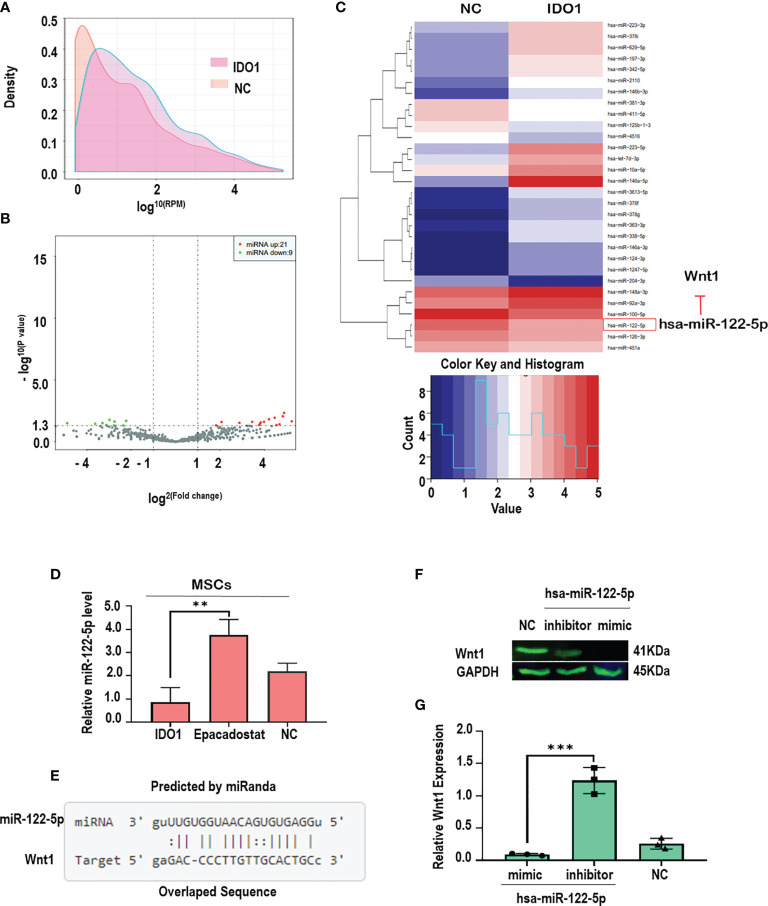
IDO1 impairs chondrogenic differentiation of MSCs by activating Wnt1/β-catenin through downregulation of miR-122-5p. **(A, B)** The density and distribution of miRNA in the total RNA obtained from MSCs-treated IDO1 compared to normal. **(C)** The screening of up-/downregulated miRNA, which shows the downregulation of miR-122-5p under the effect of IDO1. **(D)** The levels of miR-122-5p in the MSCs treated with Epacadostat compared to IDO1 and NC cells. **(E)** The target sequence of miR-122-5p in the sequence of the Wnt1 gene. **(F)** The expression of Wnt1 protein under miR-122-5p mimics compared to miR-122-5p inhibitor. **(G)** qPCR confirmative experiment for Wnt1 expression under miR-122-5p mimics compared to inhibitor in the MSCs. ***p < 0.001, and **p < 0.01 *vs* inhibitor group.

In order to explore the molecular mechanism by which IDO1 promotes the activation of Wnt1 in the stimulated MSCs, miRNA sequencing under the effect of IDO1 was performed. The results as seen in **Figures 6A–C** presented that IDO1 downregulated miR-122-5p as seen in **Figure 6D**. Further, the tested levels of miR-122-5p in the culture confirmed the effect of IDO1 on the miR-122-5p, while IDO1 inhibitor—Epacadostat—increased the expression of miR-122-5p. Importantly, the downregulation of miR-122-5p was reported to target Wnt1 in melanoma as found in miRanda database that presented Wnt1 as a perfect target for miR-122-5p, as seen in **Figure 6E** (see below) (http://mirtarbase.mbc.nctu.edu.tw/php/detail.php?mirtid=MIRT006421).

The interaction between miR-122-5p and Wnt1 in MSCs was confirmed using mimic-miR-122-5p that significantly inhibited the expression of Wnt1 as seen in **Figures 6F, G**, while miR-122-5p inhibitor showed significant expression of Wnt1 in the MSCs. These results clearly evidenced that IDO1 promotes the expression of Wnt1 through downregulation of miR-122-5p, which increases the expression of β-catenin through phosphorylation of GSK3β. Hence, the mechanism by which IDO1 impairs chondrogenic differentiation of MSCs has been successfully elucidated.

## Discussion

As known, MSCs-based therapy becomes an attractive solution for treating OA (19). New reports discussed some challenges of MSCs-based therapy for OA including the age, lesions, and severity of OA (20). However, longtime there are no clear reasons for the slowdown of MSCs differentiation and calcification in the joints of OA (21–23). Hence, this study explores for the first time the relation between the overexpression of IDO1 in the joint of OA and the low-quality response of MSCs-based therapy. The overexpression of IDO1 in the synovial fluid of OA patients has been reported by several studies (10, 14). Further studies suggested the involvement of IDO1 in the immunopathogenesis of OA (11, 12, 15–17, 24). However, the effect of IDO1 on chondrogenesis remains uncertain. This study explores a new function of IDO1 in which it works to slow down cartilage regeneration and impairing of MSCs chondrogenic differentiation. Further, this study provides evidence to the potential efficacy of IDO1 inhibitor combined with MSCs in the treatment of cartilage degeneration in the OA animal model. Furthermore, the molecular mechanism by which IDO1 impairs chondrogenic differentiation of SF-MSCs was elucidated. Importantly, this study presented a promising effect of Epacadostat to promote chondrogenic differentiation of MSCs and prevent cartilage degeneration. Hence, further studies to explore the effect of IDO1 on the biology of chondrocytes are required because it was noticed that IDO1 enhances chondrocyte apoptosis.

Previous studies reported that the inhibition of IDO1 can increase inflammation through Th1, Th17, or B lymphocytes, which are expected to enhance arthritis (13, 14), but the effect of IDO1 inhibition on the cartilage and chondrogenesis remains to be explored. Further, the real mechanism or function IDO1 could play in the synovial fluid of OA patients hasn’t been explained. Here, we found that IDO1 inhibits the differentiation of MSCs into chondrocytes through activating the signaling of the Wnt1/β-catenin pathway, which inhibits the activation of Sox9 and COL2A1. We found that IDO1 downregulates miR-122-5p and thus promotes the Wnt1/β-catenin pathway to inhibit MSCs chondrogenic differentiation. Though, the effect of the Wnt1/β-catenin pathway on chondrogenesis and OA progression has been reported (25), the connection between IDO1 overexpression and the activation of Wnt1/β-catenin pathway in the MSCs is explored for the first time in this study. Previous reports proved that the loss of β-catenin promotes chondrogenic differentiation (26, 27). In addition, this study proved that β-catenin prevents the expression of Sox9 in the stimulated MSCs by antilocalization of Sox9 protein. Thus, β-catenin plays a negative role in the progression of chondrogenesis (28–30). But the effect of IDO1 on the expression of β-catenin is still unclear. Therefore, we investigated the upstream molecules of β-catenin in the chondrogenic stimulated MSCs. Importantly, IDO1 showed significant inhibition to GSK3β. The analysis of GSK3β phosphorylation presented that IDO1 enhanced the phosphorylation of GSK3β. As known, the increase of GSK3β expression usually induces degradation of β-catenin (31, 32), which enhances cartilage formation. In this study, we observed that IDO1 significantly induces the phosphorylation of GSK3β in the MSCs, leading to an increase in the accumulation of β-catenin, which inhibits the expression of Sox9. Further, our analysis of how IDO1 enhances the phosphorylation of GSK3β leading to increase the accumulation of β-catenin revealed that IDO1 downregulates the expression of miR-122-5p and subsequently increases the expression of Wnt1 signaling that promotes the phosphorylation of GSK3β, leading to an increase in the accumulation of β-catenin, and then inhibits the expression of Sox9 and chondrogenic differentiation. Furthermore, the analysis of miRNA in the chondrogenic stimulated MSCs presented that there are some other miRNAs that have been affected by IDO1, but the link between those miRNAs and Wnt1/β-catenin signaling is absent; only miRNA-122-5p can target Wnt1 successfully. Hence, the downregulation of miR-122-5p under the effect of IDO1 is the key link between IDO1 and the activation of Wnt1/β-catenin signaling pathway in the stimulated MSCs. Moreover, though the relation between Wnt1 and miR-122-5p was reported in the melanoma (33), the connection between IDO1 and miR-122-5p/Wnt1 in chondrogenic stimulated MSCs is reported for the first time in this study. Therefore, this study explores a new function of IDO1 in the synovial fluid of OA patients by which IDO1 impairs the differentiation of MSCs to mature chondrocytes; hence, IDO1 could be a promised target in the treatment of OA.

## Conclusion

To the best of our knowledge, this is the first study that demonstrates the pathological function of IDO1 in the synovial fluid of OA patients. IDO1 impairs chondrogenic differentiation of MSCs and slows down cartilage regeneration through regulating miR-122-5p/Wnt/β-catenin signaling that inhibits the activation of Sox9 and COL2A1. Animal model experiments presented a promising method to improve the outcomes of MSCs-based therapy for OA patients through combining the use of IDO1 inhibitor (Epac) with MSCs to promote cartilage regeneration. This study contributes to explain a clear reason of challenges facing MSCs-based therapy in OA. The use of IDO1 inhibitor with MSCs could improve the outcomes of clinical trials. Further, this study elucidated that IDO1 could be a promising target in the treatment of OA.

## Data Availability Article

The authors confirm that all the data related to this research have been included in the manuscript and **Supplementary Material**. miRNA transcriptome sequencing data are available by the link https://www.ncbi.nlm.nih.gov/sra/PRJNA758741.

## Ethics Statement

The studies involving human participants were reviewed and approved by the National Ethical Committee of Shenzhen Second People’s Hospital. The patients/participants provided their written informed consent to participate in this study. The animal study was reviewed and approved by the National Ethical Standards and Guidelines for Animal Use of Shenzhen Second People’s Hospital Ethical Committee.

## Author Contributions

MA: conceptualization, methodology, data curation, formal analysis, writing—reviewing and editing. RH and LD: manuscript revision, formal analysis, and editing. DZ: revising. OH, WL, and DW: supervision and final approval. All authors contributed to the article and approved the submitted version.

## Funding

This work is supported by the International Science and Technology Cooperation Project of Guangdong Province 2019, China (1035043); Shenzhen Municipal Human Resources and Social Security Bureau, the first batch of post-doctoral research grants (1038051); National Natural Science Foundation of China (No. 81772394, No. 81972116, No. 81972085); a key clinical discipline of Guangdong Province, Orthopaedics (No. 2000005).

## Conflict of Interest

The authors declare that the research was conducted in the absence of any commercial or financial relationships that could be construed as a potential conflict of interest.

## Publisher’s Note

All claims expressed in this article are solely those of the authors and do not necessarily represent those of their affiliated organizations, or those of the publisher, the editors and the reviewers. Any product that may be evaluated in this article, or claim that may be made by its manufacturer, is not guaranteed or endorsed by the publisher.
