# Supplementary material for: Indoleamine 2, 3 Dioxygenase 1 Impairs Chondrogenic Differentiation of Mesenchymal Stem Cells in the Joint of Osteoarthritis Mice Model
Source: Front Immunol. 2021 Dec 8;12:781185. doi: 10.3389/fimmu.2021.781185 (PMC8693178; doi:10.3389/fimmu.2021.781185)
Supplement: Supplementary file 1 [file DataSheet_1.docx]

**Indoleamine 2, 3 dioxygenase 1 Impairs Chondrogenic differentiation of Mesenchymal Stem Cells in the Joint of Osteoarthritis Mice Model**

# Supplementary data


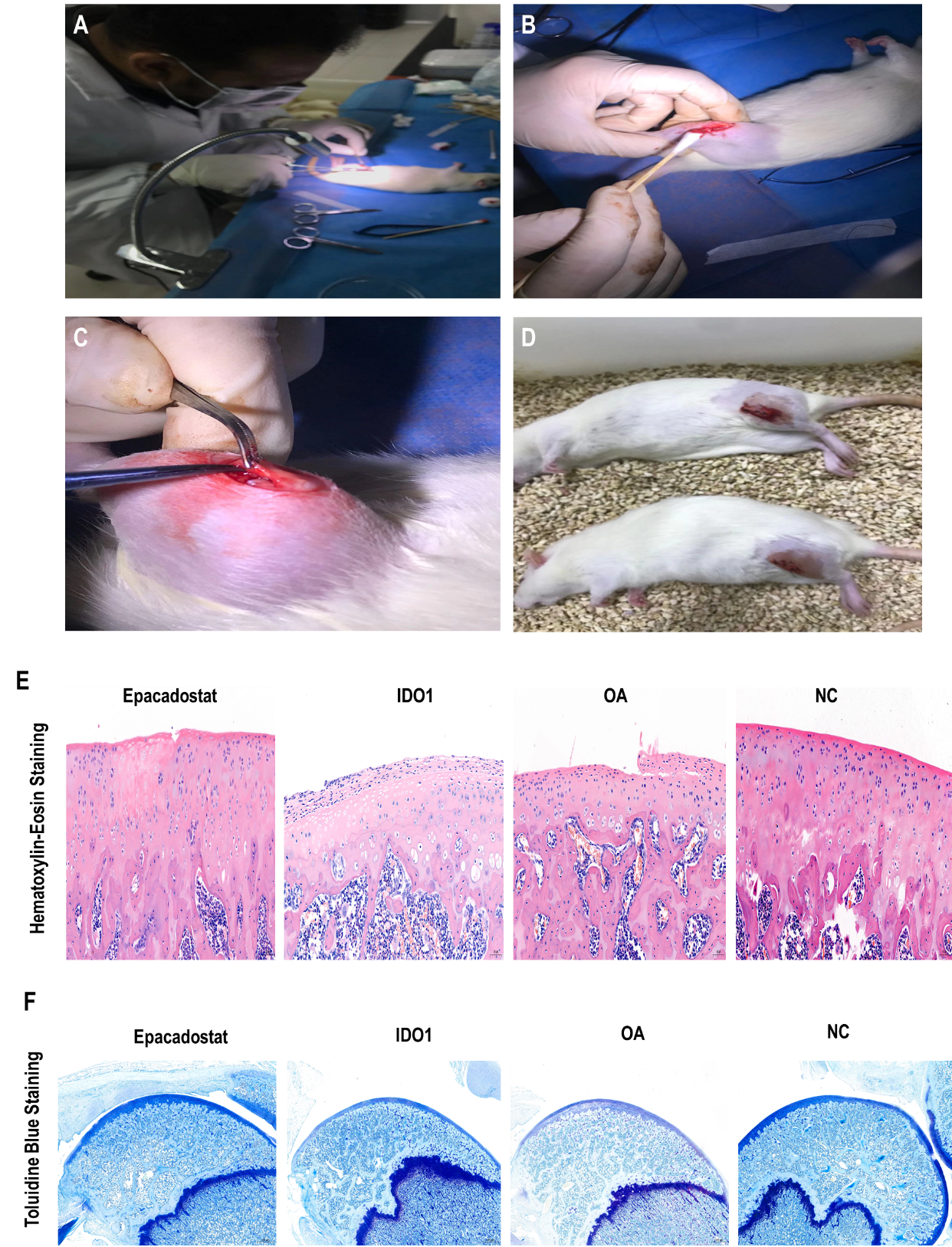


Si.Fig.1: The establishment of OA in the knee of SD rats using a meniscectomy method. (A-D) the operation process of cutting meniscus according to the operation guidelines. (E) HE staining of the joint after one month of the operation, which presented the initiation of cartilage degeneration in OA group and IDO1+MSCs group. (F) Toluidine blue staining presented significant changes in the thickness of cartilage layer in the OA and IDO1+MSCs groups compared to other tested groups.


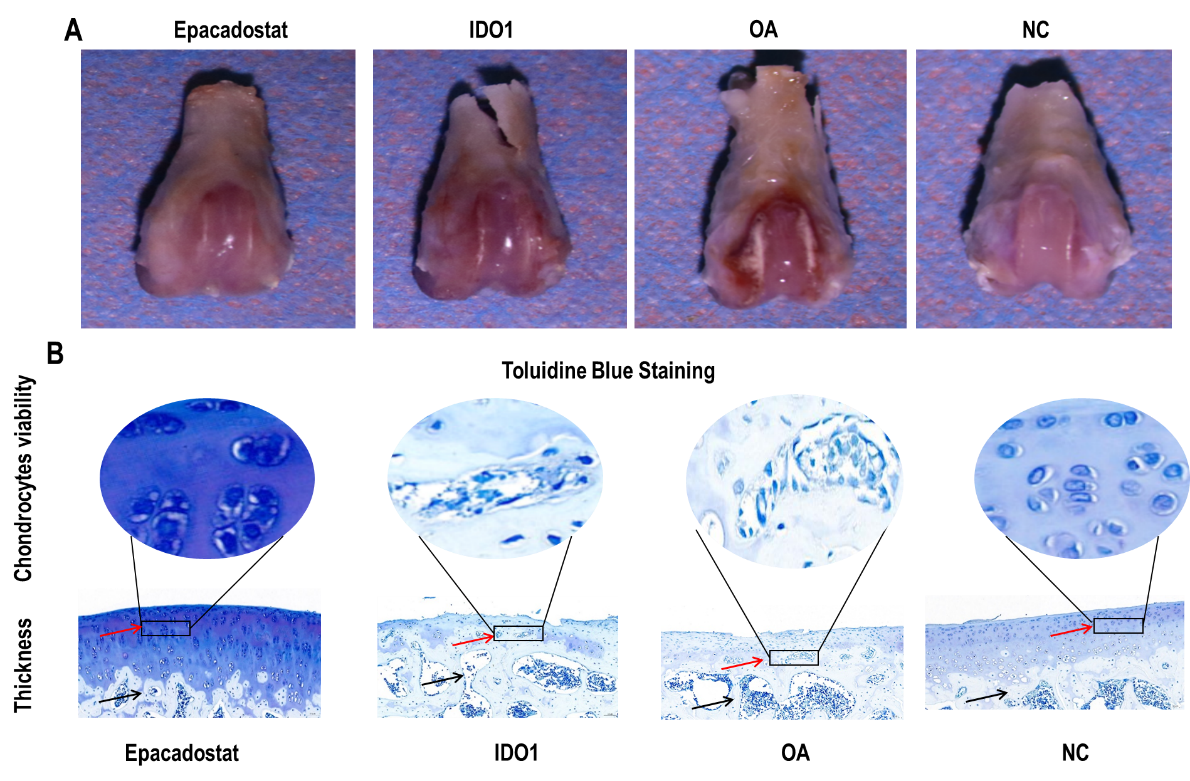


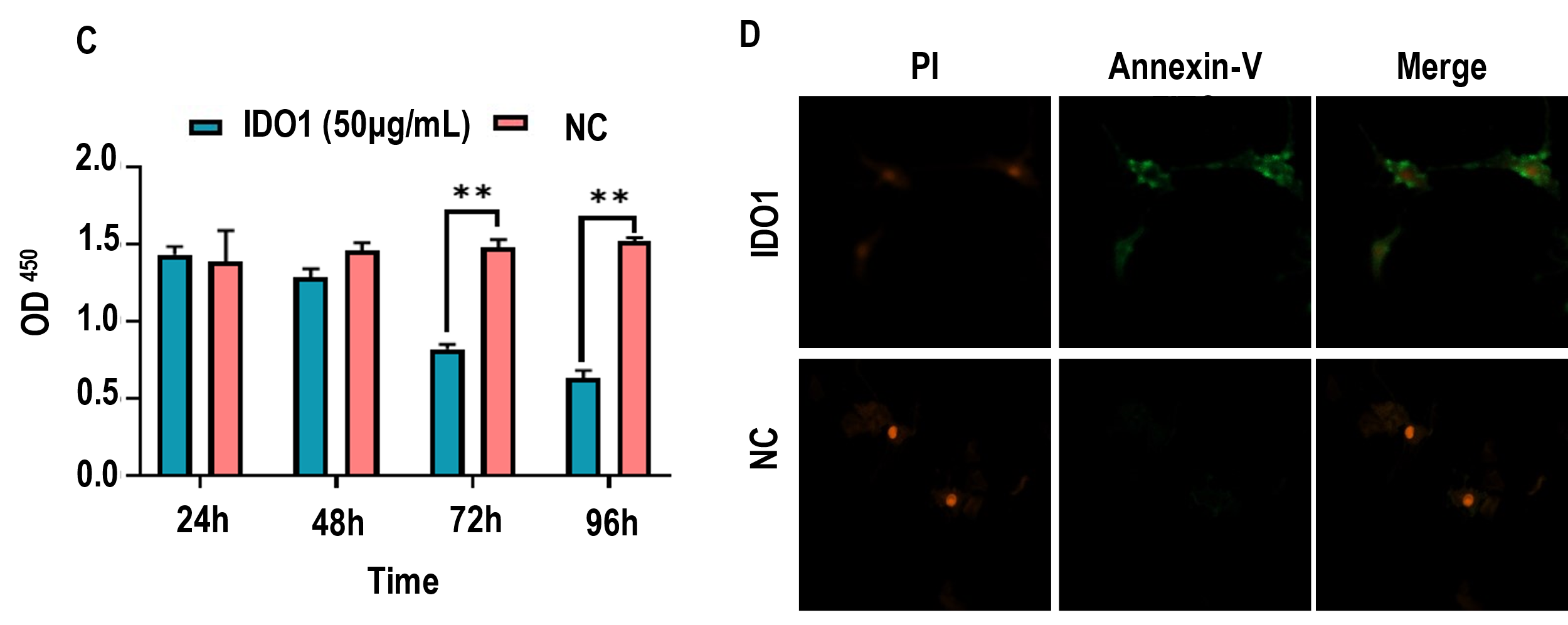


Si.Fig.2: MSCs mixed with Epacadostat promoted cartilage regeneration in the OA animal model. (A) MSCs mixed with Epacadostat enhanced cartilage regeneration compared to IDO1 mixed cells that showed no improvements in the cartilage and high degenerative activity. (B) MSCs mixed with Epacadostat significantly enhanced the number of mature chondrocytes in the cartilage tissue compared to IDO1 that showed apoptosis of chondrocytes in the cartilage. (C) the apoptotic effects of IDO1 on the chondrocytes compared to normal control using cck8 assay. (D) the apoptotic effects of IDO1 on the chondrocytes compared to normal control using Annexin-V assay

##
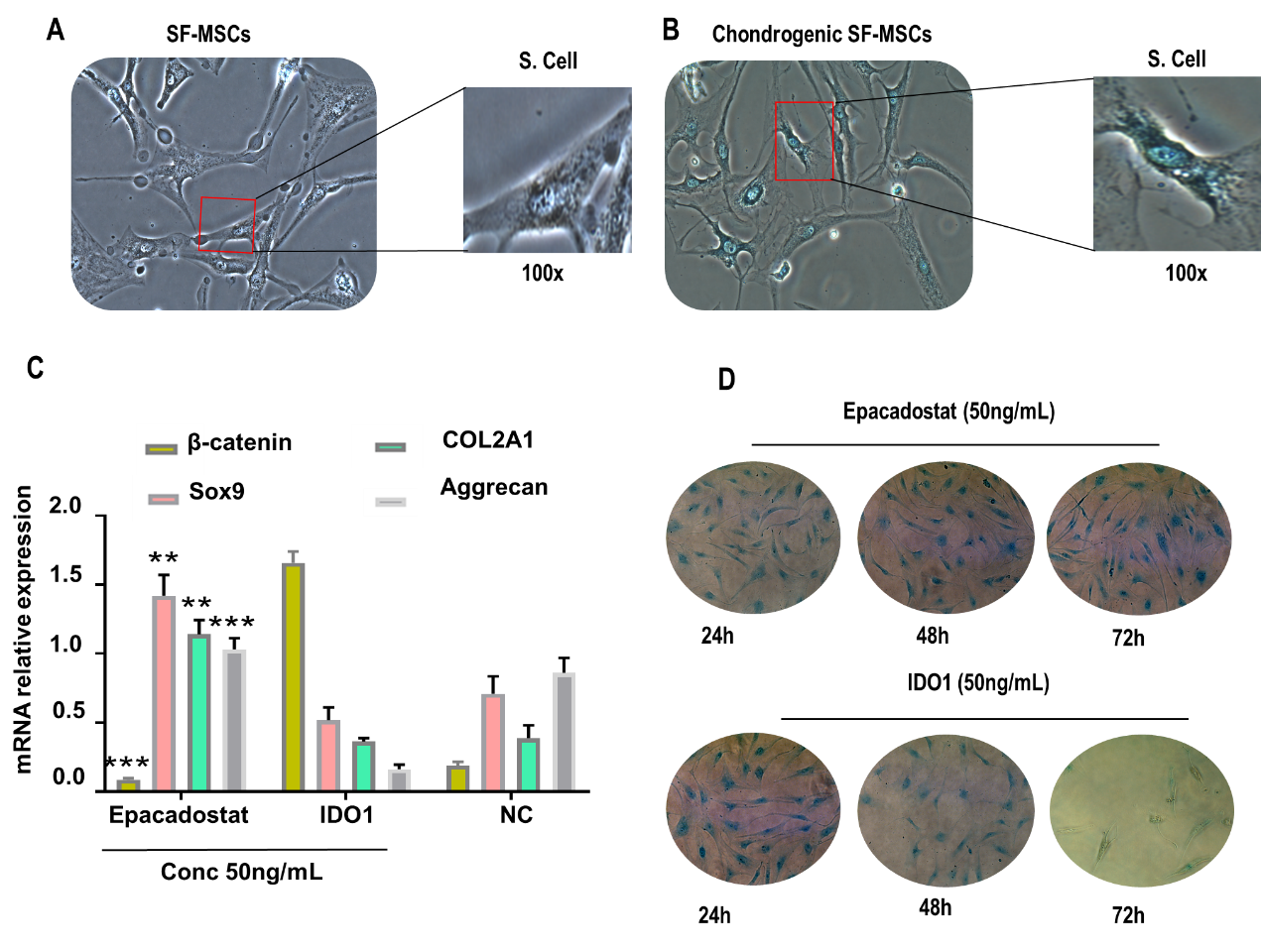


## Si.Fig.3: Characterization of chondrogenic MSCs. (A) a photography of non-chondrogenic MSCs that showed negative for alcian blue staining. (B) a photography of chondrogenic SF-MSCs that showed positive staining of alcian blue. (C) the expression of chondrogenic signaling Sox9, COL2A1, Aggrecan, and β-catenin under the effect of Epacadostat compared to IDO1. (D) alcian blue staining to explore the accumulation of proteoglycan that indicating chondrogenic differentiation. Showed that stimulated MSCs was significantly differentiated under the effect of Epacadostat to chondrocytes compared to IDO1 (*p*< 0.01). ****p* < 0.001, and ** *p* < 0.01 *vs* NC group


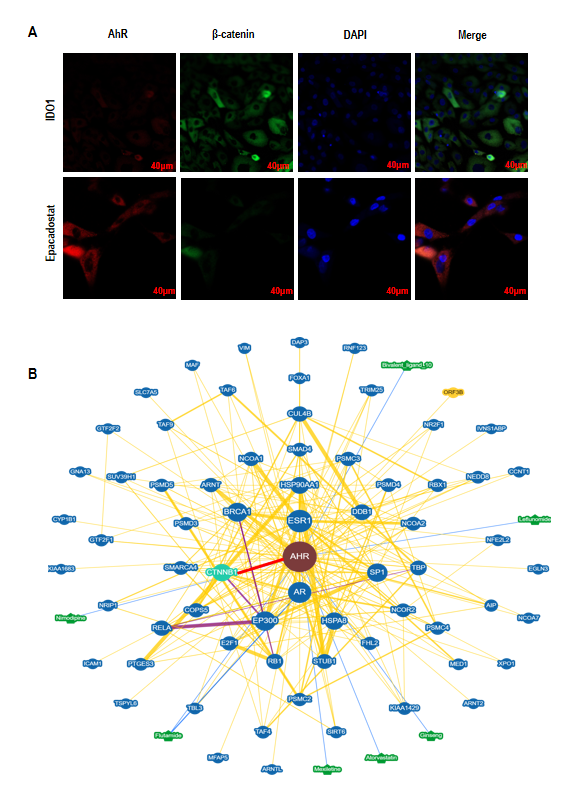


**Si.Fig.4**: the relation between AhR and Epacadostat in the MSCs. (A) Epacadostat enhances AhR expression while reduces the expression of β-catenin, meanwhile, IDO1 reduced the expression of AhR and enhances the expression of β-catenin in the MSCs. (B) the bioinformatics prediction of protein interaction between AhR and β-catenin (CTNNB1) using the database of (String-db.org).


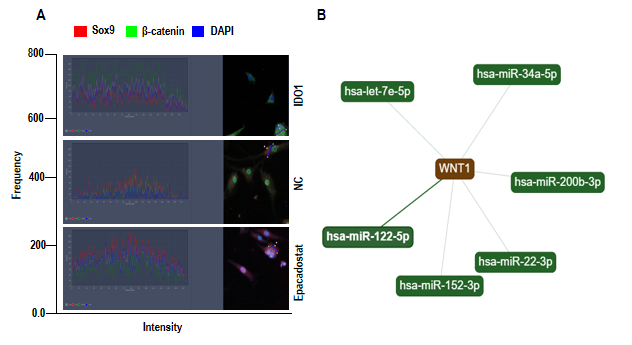


**Si.Fig.5**: the relation between IDO1 and β-catenin in the MSCs. (A) the intensity of anti-localization between Sox9 and β-catenin under the effect of IDO1 compared to Epacadostat. (B) Bioinformatics analysis of the interaction between Wnt1 gene and miR-122-5p.

# Supplementary Table (1): list of predicted sequences of primers forward and reverse for qPCR

| Gene name | NCBI Gene ID | Sequence 5' -> 3' | |
| --- | --- | --- | --- |
|  |  | Forward | Reverse |
| IDO1 | 3620 | GCCAGCTTCGAGAAAGAGTTG | ATCCCAGAACTAGACGTGCAA |
| β-catenin | 1499 | AAAGCGGCTGTTAGTCACTGG | CGAGTCATTGCATACTGTCCAT |
| Sox9 | 6662 | AGCGAACGCACATCAAGAC | CTGTAGGCGATCTGTTGGGG |
| Ihh gene | 3549 | AACTCGCTGGCTATCTCGGT | GCCCTCATAATGCAGGGACT |
| Collagen II | 1280 | TGGACGATCAGGCGAAACC | GCTGCGGATGCTCTCAATCT |
| Aggrecan | 176 | ACTCTGGGTTTTCGTGACTCT | ACACTCAGCGAGTTGTCATGG |
| GSK3α | 2931 | GGAAAGGCATCTGTCGGGG | GAGTGGCTACGACTGTGGTC |
| GSK3β | 2932 | GGCAGCATGAAAGTTAGCAGA | GGCGACCAGTTCTCCTGAATC |
| APC | 324 | AAAATGTCCCTCCGTTCTTATGG | CTGAAGTTGAGCGTAATACCAGT |
| AhR | 196 | ACATCACCTACGCCAGTCG | CGCTTGGAAGGATTTGACTTGA |
| Wnt1 | 7471 | CGATGGTGGGGTATTGTGAAC | CCGGATTTTGGCGTATCAGAC |
| GAPDH | 2597 | GGAGCGAGATCCCTCCAAAAT | GGCTGTTGTCATACTTCTCATGG |

#

# Supplementary Table (2): list of predicted siRNA sequences that have used in this study

| Gene Name | siRNA | siRNA Sequence | |
| --- | --- | --- | --- |
|  |  | Sense 5'- 3' | Antisense 5' - 3' |
| β-catenin | 1 | CCUUCACUCAAGAACAAGUTT | ACUUGUUCUUGAGUGAAGGTT |
| β-catenin | 2 | GCUCAUCAUACUGGCUAGUTT | ACUAGCCAGUAUGAUGAGCTT |
| β-catenin | 3 | GUCAACGUCUUGUUCAGAATT | UUCUGAACAAGACGUUGACTT |
| IDO1 | 1 | CCCUGUGAUAAACUGUGGUUU | UUGGGACACUAUUUGACACCA |
| IDO1 | 2 | GUGAUAAACUGUGGUCACUUU | UUCACUAUUUGACACCAGUGA |
| IDO1 | 3 | CUGUGAUAAACUGUGGUCAUU | UUGACACUAUUUGACACCAGU |
| Negative Control |  | UUCUCCGAACGUGUCACGUTT | ACGUGACACGUUCGGAGAATT |

Online viability of original data

**ELISA**

[**https://www.jianguoyun.com/p/DVuXLbgQvYTnCRjH1ooE**](https://www.jianguoyun.com/p/DVuXLbgQvYTnCRjH1ooE)

**H&E Staining**

[**https://www.jianguoyun.com/p/DQEasdUQvYTnCRjJ1ooE**](https://www.jianguoyun.com/p/DQEasdUQvYTnCRjJ1ooE)

**Safranine O green**

[**https://www.jianguoyun.com/p/DV9MROYQvYTnCRjC1ooE**](https://www.jianguoyun.com/p/DV9MROYQvYTnCRjC1ooE)

**qPCR**

[**https://www.jianguoyun.com/p/Da6q9ZkQvYTnCRjN1ooE**](https://www.jianguoyun.com/p/Da6q9ZkQvYTnCRjN1ooE)

**Immunofluorescent assay**

[**https://www.jianguoyun.com/p/DaliIuQQvYTnCRjR1ooE**](https://www.jianguoyun.com/p/DaliIuQQvYTnCRjR1ooE)

**Cell images**

[**https://www.jianguoyun.com/p/DSZmMWsQvYTnCRjS1ooE**](https://www.jianguoyun.com/p/DSZmMWsQvYTnCRjS1ooE)

**Western blot**

[**https://www.jianguoyun.com/p/DZRgXusQvYTnCRjT1ooE**](https://www.jianguoyun.com/p/DZRgXusQvYTnCRjT1ooE)

**Bioinformatic links**

[**https://www.jianguoyun.com/p/DU2YoS0QvYTnCRjc1ooE**](https://www.jianguoyun.com/p/DU2YoS0QvYTnCRjc1ooE)

**Flowcytometry**

[**https://www.jianguoyun.com/p/Da-QhjMQvYTnCRiujY4E**](https://www.jianguoyun.com/p/Da-QhjMQvYTnCRiujY4E)

**Apoptosis**

[**https://www.jianguoyun.com/p/DVtYiV4QvYTnCRjljJgE**](https://www.jianguoyun.com/p/DVtYiV4QvYTnCRjljJgE)
